# Parametric Cognitive Modeling of Information and Computer Technology Usage by People with Aging- and Disability-Derived Functional Impairments

**DOI:** 10.3390/s16020266

**Published:** 2016-02-22

**Authors:** Rebeca I. García-Betances, María Fernanda Cabrera-Umpiérrez, Manuel Ottaviano, Matteo Pastorino, María T. Arredondo

**Affiliations:** Life Supporting Technologies (LifeSTech), Superior Technical School of Telecommunication Engineers, Universidad Politécnica de Madrid (UPM), Avenida Complutense n° 30, Ciudad Universitaria, Madrid 28040, Spain; rgarcia@lst.tfo.upm.es (R.I.G.-B.); mottaviano@lst.tfo.upm.es (M.O.); mpastorino@lst.tfo.upm.es (M.P.); mta@lst.tfo.upm.es (M.T.A.)

**Keywords:** virtual user models, parametric cognitive models, ICT health tools, multisensorial platform, disabilities-friendly products design, user needs, personalization, accessibility

## Abstract

Despite the speedy evolution of Information and Computer Technology (ICT), and the growing recognition of the importance of the concept of universal design in all domains of daily living, mainstream ICT-based product designers and developers still work without any truly structured tools, guidance or support to effectively adapt their products and services to users’ real needs. This paper presents the approach used to define and evaluate parametric cognitive models that describe interaction and usage of ICT by people with aging- and disability-derived functional impairments. A multisensorial training platform was used to train, based on real user measurements in real conditions, the virtual parameterized user models that act as subjects of the test-bed during all stages of simulated disabilities-friendly ICT-based products design. An analytical study was carried out to identify the relevant cognitive functions involved, together with their corresponding parameters as related to aging- and disability-derived functional impairments. Evaluation of the final cognitive virtual user models in a real application has confirmed that the use of these models produce concrete valuable benefits to the design and testing process of accessible ICT-based applications and services. Parameterization of cognitive virtual user models allows incorporating cognitive and perceptual aspects during the design process.

## 1. Introduction

In recent years, most of the world’s developed countries have been experiencing significant demographic changes, including the rising average age of their populations [1]. According to the 2013 Eurostat yearbook [2], by January 2012 the number of people aged 65 and above amounted to 26.8% of the working-age population in Europe (EU-27). It is expected that these changes in European demographic distribution will be accentuated during the coming decades due to consistent low birth rates and increasing life expectancy prevalent in the region. Eurostat foresees that by the year 2060 there will be in Europe less than two people of working age (15 to 64) for every elderly person (65 or older) [3].

The aging of the general population has direct implications for the future of Information and Communication Technologies (ICT) development, and poses significant challenges to their widespread use. The percentage of the European (EU-27) population aged 65 to 74 using the Internet increased between the years of 2005 and 2010 from 5% to 17% for daily use, and from 10% to 25% for weekly use [3]. These percentages are expected to increase further in the coming years, mainly because of the wave of computer technology adoption dynamics represented by the increase of the population of older individuals who make intensive use of computers [1,4]. Additionally, the prevailing ageing tendency of European demography as a side effect produces an increase in the proportion of European citizens affected by significant disabilities. There are presently about 80 million people in Europe with disabilities, representing over 15% of its total population. The European Disability Forum indicates that one out of four Europeans has a family member with a disability [5].

As ICT solutions increasingly become an indispensable part of the lives of elderly people, as well as those with disabilities in general, it is of paramount importance for researchers and developers of ICT solutions to devise and adopt new design methodologies that devote special attention to these people’s particular needs and requirements [1], as well as reduce the generation gap with ICT products and integrate older people and people with disabilities into the information society. A broader integration would translate into abundant and significant benefits, not only to these individuals themselves, but also to society as a whole [6]. These people’s ICT needs differ considerably from those of a normal or younger user. People’s functional impairments derived from disabilities come in a wide variety of manifestations and generally differ broadly from one case to another. The severity of any preexisting disability likely will increase with age. On the other hand, previously healthy people will certainly develop aging-related functional impairments as they approach old age [1,4].

ICT designers and developers generally do not have adequate knowledge about the different conditions and cognitive states that people with aging- and disability-derived functional impairments normally experience, nor do they have sufficient expertise about the difficulties and impairments these people face when using ICT services or applications.

One of the main prerequisites of designers and developers, either designing for ICT environments/applications or not, is that interactions of end users must be multimodal and adaptive according to user preferences, disabilities and changing daily environments. In principle, different modalities can be used concurrently so as to increase the accessibility of available information or, alternatively, to present the same information in different contexts, or, redundantly, to address different interaction channels. Nowadays, these principles have been used in many different studies and proposals for design methodologies [7,8].

### 1.1. User Modeling

Researchers and developers nowadays are incorporating new paradigms into their repertoire of service personalization tools, in addition to users’ self-customization. These new paradigms gravitate around the perspective of an inclusive and universal design concept to satisfy the needs and requirements of particular groups of users, such as the elderly and people with disabilities. The use of inclusive and universal design techniques and tools in an adequate manner could help to provide solutions to the particular problems that these users have to confront when using today’s ICT systems and applications [1,9]. Some of these problems are related to usability issues, such as: functional limitations, behavioral and cognitive limitations, reduced capabilities, *etc.*, which could strongly influence user acceptance [10,11]. Both of these groups of people have needs and concerns different from those of general users, because of their cognitive and physical impairments, stemming from aging or from some previously existing disability. These needs and concerns are often ignored during the design and development process of ICT solutions [11]. Therefore it is imperative to understand: (a) how the capabilities and conditions of these groups of users differ from those of other users; (b) what are the implications and usability problems derived from those differences; (c) how these groups of users react towards systems; and (d) how the systems can be made more usable for them [1].

User-centered design (UCD) is a modern Human Computer Interaction (HCI) methodology that is based on inquires, assumptions, and analysis of users’ needs, desires, and limitations during the design process of ICT solutions [11]. The principal objective of UCD is to include the user into the design process through the use of different user modeling techniques that typify a group of users, such as: feature-based, content-based, case-based, scenario and goal-oriented, knowledge-based, demographic modeling, *etc.* [12,13].

Various studies and developments have been carried out to overcome several of the technological barriers faced by users affected by specific impairments or limitations, taking into account their particular characteristics and needs [11,14,15,16]. In 2001 Fischer [17] proposed to specifically classify users into different kinds, with respect to particular needs and characteristics, and within various environments. But a classification of users merely according to their expertise and knowledge (e.g., novel, intermediate, and expert) was found not to be enough. In 2002 Gregor *et al.* [16] presented a discussion about the particular issues involved in the design process of ICT solutions specifically geared to elderly users. They identified some capabilities that are relevant and imperative to be considered during the design process, such as: physical, sensory, and cognitive abilities; learning capacity; memory problems; environmental factors; and accumulated experience [16].

Cognitive architectures are theories for simulating and understanding how human mind works. They use cognitive psychology known theories and their assumptions, about working memory, attention, decision making, *etc.*, to implement them in a computer program that makes knowledge representations to reproduce human thinking. ACT-R [18] is perhaps the most popular and best known cognitive architecture which provides a conceptual framework for creating models of how people perform tasks. ACT-R’s constructs reflect assumptions about human cognition, based on numerous facts derived from psychology experiments. Different studies have used this cognitive architecture to simulate user’s cognitive functions through a computational cognitive model individualized to the specific user [19], and simulate the progression of cognition disabilities during the execution of activities of daily living [20].

In 2004 Cooper [21] introduced the concept of “persona” by stating in this context that “personas are not real people, but they represent them throughout the design process. They are hypothetical archetypes of actual users.” This definition allows distinguishing between different user groups during the design and development of an adaptive ICT user interface domain, focusing on the user’s needs instead of on user’s capabilities. Working with the “persona” concept throughout the entire design process helps researchers and designers understand and effectively take into account user’s needs. Other researchers and developers also have used this concept and methodology to design ICT systems that are more aware of the user abilities and limitations [21,22,23]. Also some studies propose a combination of the “persona” concept with other UCD methodologies such as: goal and scenario modeling [24]. In 2007 Aoyama [24] proposed a Persona-Scenario-Goal (PSG) methodology as an extension of the persona-scenario methodology.

Despite the potential benefits of UCD methodologies their use, in particular for health technologies, has not received considerable attention [11]. This affirmation is true also in the context of applications for aged patients. Although literature provides low guidance and directions to involve older people during the design and development process, some studies have been carried out taking into account this population segment [11,12].

ICT applications and services usually were unable to guarantee a comfortable interaction for most types of these users. An official medically-oriented study that provided guidance regarding special user capabilities and conditions ensuing from aging or disability derived functional impairments was published in the 80’s by the World Health Organization (WHO). This classification was first called the International Classification of Impairments, Disabilities, and Handicaps (ICIDH). In 2001, a new version called the International Classification of Functioning, Disability and Health (ICF) was approved by the WHO World Health Assembly [25,26]. The ICF classification defined and classified health and other health-related domains. The document included the concepts of “health” and “disability,” the environmental factors involved, their impact, a classification of every function state associated with health (e.g., diseases, disruptions, injuries, traumas, *etc.*), and a common framework to allow all conditions to be compared using common metrics. The ICF documentation represents an essential starting reference for researchers, designers, and developers to identify user capabilities and conditions that shape the settings of the interaction processes [22,27].

Methodological processes of user-centred design are recently moving towards cognitive support type of implementation [28]. Eberle *et al.* [10] have emphasized the convenience of introducing cognitive user processes into user modeling techniques. Their argument is that it should be possible to “state adaptation methods and techniques in terms of a cognitive aid vocabulary”. The use of such cognitive tools and techniques frees the designer and developer from having to make speculative suppositions or dubious estimates about medical conditions and other related issues.

Nowadays, assistive technologies are trying to apply UCD methodologies and techniques to understand user’s perspective and implicate, in their solutions, user’s goals, abilities, and needs, as well as user’s context or scenario [29].

### 1.2. VERITAS Project Overview

The VERITAS [30] project focused on the creation of an open framework for providing built-in accessibility support at all the stages of the development of mainstream ICT and non ICT technologies, including specification, design, development, and testing. The goal is to introduce simulation-based and virtual reality testing at all stages of assistive technologies design and development different application contexts (e.g., automotive, smart living spaces, workplace and infotainment). The goal is: (1) to ensure that future products and services are being systematically designed for all people including those with disabilities and functional limitations as well as older people; and (2) provide product/software developers with the necessary tools for exploring new concepts, designing new interfaces and testing interactive prototypes that will inherit universal accessibility features, including compatibility with established assistive technologies.

The main innovation of the VERITAS project regarding virtual user modelling was the introduction and use of a multisensorial platform for the training of parameterized user models based on real user measurements, in real testing conditions. The platform provides the technological infrastructure for the capture of user motion in each application contexts. Also, the multisensorial platform is fully parameterized and adapted to the VERITAS application areas and is used to capture real user feedback while executing a number of tasks that will be mapped in the VERITAS virtual user models. The components of the multisensorial platform are the followings:

- VIDEO SENSING: a motion detection system based on three cameras positioned all around the subject.
- WEARABLE: constituted by a set of systems that can be used to collect data from about movements of several body parts of body or joints, such as: gloves, knee electro-goniometers and accelerometers.
- MOTION TRAKING: using commercial stereoscopic cameras to analyze gait activity, identify specific irregular walking patterns and to extract parameters like height, step and stride length and width, step asymmetries, cadence, body oscillation (width shift) during gait as well as hip, knee and hand range of motion.
- ENVIRONMENTAL SENSORS: to capture and analyze user interaction with objects and interfaces in order to complement the vision given by the wearable sensors and cameras system.

The present study aims to define parametric cognitive virtual user models that describe interaction and usage of ICT by people with aging- and disability-derived functional impairments and evaluate their use during the design process and accessibility evaluation of ICT applications. The article is organized as follows: Section 2 describes the methodological phases and the approach used to define each part of the parametric Cognitive Virtual User Model; Section 3 and Section 4 describe the Results in terms of the defined framework and the evaluation through simulation of the models. It specifically shows the parameters and affected tasks defined, the structures and representations through the ontologies, the evaluations results of the final parameterized Cognitive Virtual User Model in a remote health monitoring application and an analytic discussion of the results; Finally Section 5, Discussion and Conclusions presents conclusions, a comparison to prior works, the principal contributions and the limitations and future work of the study.

## 2. Study Design and Methodology

### 2.1. Study Description

The present study’s main objective aims at defining parametric cognitive models to describe the interaction and usage of ICT by people affected by aging- and disability-originated functional impairments and cognitive states. It also intends to provide a foundation for generating a large pool of virtual users to act as subjects of the test-bed to be used during all stages of simulated ICT health products and systems design, including user accessibility design and evaluation. One of the main innovations of the project related to virtual user modelling is the use of a multisensorial training platform (previously described in Section 1.2) to train the virtual parameterized user models based on real user measurements in real conditions. Special sensors, such as face monitoring cameras, driver monitoring sensors, wearable sensors for body motion analysis, motion trackers, gait analysis sensors, and environmental sensors, were used for data capture. Tables were used to make the parametric representation of the user models in a readable format and them instantiate them with semantic ontologies. Also, an innovative mechanism of task-based analysis was used to validate, update and train the developed parameterized user models. With this, the virtual user models developed could be iteratively trained and verified by people with disabilities in real testing conditions.

### 2.2. Methodology

In order to conceive, construct and parameterize the target users’ cognitive models to be used within the VERITAS project’s accessibility design tools, the following actions were undertaken [31]:

- Identify which are the cognitive functions and their corresponding parameters (e.g., reaction time, perception, attention, working memory, *etc.*) that are relevant to each type of cognitive impairment of interest: elderly people, Alzheimer’s disease patients, Parkinson’s disease patients, and people with visual, hearing and speech impairments.
- Analyze and characterize specific target users’ needs.
- Define the recommendations, guidelines and values that will support the designers and developers’ decisions during the designing process of new ICT health products and services.
- Evaluate the final parameterized cognitive user models by simulating them in a real application.

We have used the Adaptive Control of Thought-Rational (ACT-R) [18] to address the above listed aspects of user models. This selection was done based on the characteristics of this cognitive architecture that make it the best suited architecture to model most of these user’s cognitive models for disabled and elderly users.

**Figure 1:**
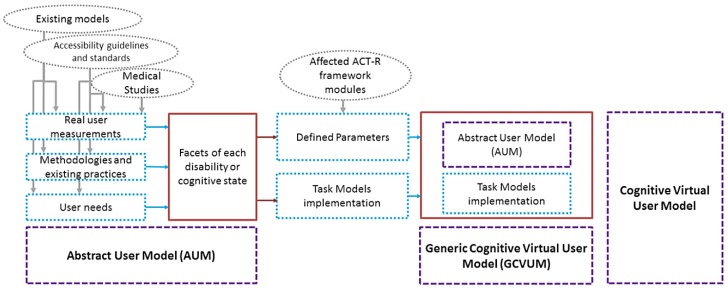
Overview of the process to create the Cognitive Virtual User Model.

The process of generating and modeling the final Cognitive Virtual User Model consisted of the following steps that are also showed in Figure 1:

1. Generation of an Abstract User Model (AUM), based on the analysis of existing models, medical studies, guidelines, real user measurements, methodologies and existing practices, user needs, as well as known accessibility guidelines and standards. The Cognitive AUM represents the different facets of each disability or cognitive state.
2. Mapping between the defined parameters and the affected ACT-R framework modules.
3. Task Models implementation to represent users while performing specific tasks and interactions.
4. Generic Cognitive Virtual User Model (GCVUM) generation, by merging the cognitive AUM with the affected tasks per disability/cognitive state.
5. Cognitive Virtual User Model generation, by instantiating the GCVUM.
6. Simulation and evaluation of the final parameterized Cognitive Virtual User Models on a real remote health monitoring application.

#### 2.2.1. Cognitive Abstract User Modeling

An Abstract User Model (AUM) is a descriptive representation of the different dimensions of a human being. Disease-related parameters and data sets included within the cognitive AUM are “abstract”, in the sense that they are independent of the actual modeling technique that will be used later to generate the Virtual Users. Functional limitations are described by abstract parameters and data. The most relevant cognitive functions and processes related to a specific cognitive disability context are collected to create a representation of the cognitive AUM in a table structure that relates the cognitive disabilities with the affected task models. The cognitive processes are divided in two major groups: basic and higher-level process groups. The basic cognitive group is composed of low-level cognitive functions such as: reaction time, attention (subdivided into selective, divided and sustained), memory (subdivided into semantic, episodic, procedural and working), and perception (subdivided into visual, auditory and haptic). The higher-level group is composed of cognitive functions that arise from the low-level functions and implies more complex cognitive processes. The included higher-level cognitive functions are: decision making, orientation, and speech and language. These cognitive functions were selected to take into account the most important functions that could be impaired or could participate in the execution of the defined tasks.

Different parameter categories are addressed for each cognitive state, such as: category, description, types, International Classification of Diseases (ICD), causes, age-relation, ICF functional limitations and cognitive response. Other parameters also addressed and compiled were: affected attributes per cognitive state, existing computational and theoretical models, and rules or possible alternative measurements.

Ontologies are used to provide powerful and growing specification of the cognitive AUM. The cognitive AUM stored in the ontology includes the type of user disability, user capabilities, user needs, characteristics from cognitive user models, physical user models, behavioral and psychological user models, guidelines and standards.

#### 2.2.2. ACT-R User Model Structure

The (quantitative) definition of parameter values that are not included in the Cognitive AUM definition is based on an examination of the existing cognitive architecture ACT-R models, a well-researched and commonly used cognitive modeling system. ACT-R provides a conceptual framework for creating models of how people perform tasks based on sets of theoretical assumptions. Although cognition is the main part of ACT-R, this cognitive architecture also represents perception and motor behavior within its different modules [32,33,34]. ACT-R makes use of a modular organization composed of eight modules: two perceptual modules (visual and aural) to receive the external stimuli from the environment; two response modules (manual and vocal) to provide interaction with the environment; and four modules (problem state, declarative, goal, and procedural) responsible for different aspects of central processing. The interaction among all these modules takes place via the procedural module. Each module is related to a function and to specific regions of the human brain [35].

Parameter values for each of the possible types of end-user (elderly and cognitive impaired in different domains) were extracted and collected through a wide literature research [36]. Values of these parameters per cognitive disability, as related to the cognitive processes, were detected and analyzed in the AUM, in order to specify possible mechanisms to be implemented within the overall architecture. Identification of possible changes to current mechanisms allows simulating the effect of cognitive impairments on embodied cognition. A complete ACT-R (sgp—set global parameters) user model structure was described, that includes all the previously defined and collected information.

#### 2.2.3. Affected Task Analysis

The initial research, done within the cognitive AUM definition and representation, determined the most relevant cognitive disabilities (basic and high-level functions). However, this information seems to be limited. A developer using a final Cognitive Virtual User Model has to be able to set up a virtual model of people affected by aging- and disability-originated functional impairments and cognitive states, and then use it to simulate different tasks specific to different application sectors. Typical sectors are Personal Healthcare and Wellbeing, Automotive, Smart living spaces, and Workplace. Accordingly, the user model has to link disability related information with information related to different tasks associated with the different sectors. The purpose of this connection is to identify how different cognitive states affect the user’s cognitive functions (reaction time, memory, attention, *etc.*), and then to extract relevant quantitative and qualitative metrics, rules and parameters.

Functional limitations and cognitive response to specific tasks are classified according to ICF classification guidelines [26]. Tasks (e.g., using PC, using mobile devices, *etc.*), subtasks (e.g., use keyboard, switch on/off cellphone, interaction with touch screen, *etc.*), primitive tasks (e.g., see, pointing, reach, push, *etc.*), and cognitive states affecting primitive tasks, were defined and linked. All the collected information related to the Personal Healthcare and Wellbeing sector was analyzed and merged into the Cognitive AUM description.

#### 2.2.4. Generic Cognitive Virtual User Model

Generic Cognitive Virtual User Model (GCVUM) generation follows a user-centric approach and represents the AUM and existing standards, metrics and guidelines using Web Ontology Language (OWL) ontologies [37]. Additionally, a multi-sensorial platform designed and implemented within the VERITAS project was used to sense the needs of real users with disabilities, by measuring their behavior in simulated environments.

**Figure 2:**
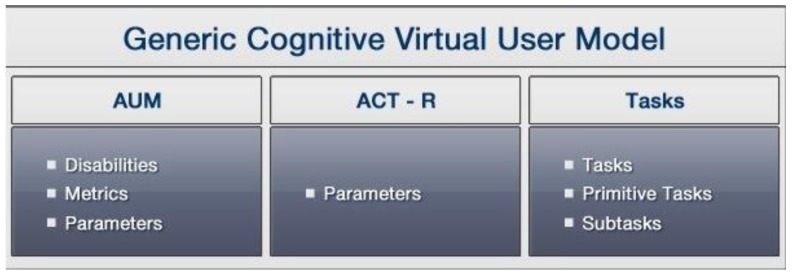
Information needed to generate the Generic Cognitive Virtual User Model.

The GCVUM linked specific disability information with information related to different tasks, in order to set up the final parameterized Cognitive Virtual User Model of a disabled or elderly person to be used to simulate different tasks. This is accomplished by merging together the information contained in the Cognitive AUM, the ACT-R parameter values, the collected data about how a specific cognitive state could be affected, and the affected task models. Figure 2 presents the described structure with the merged information necessary to create the GCVUM.

All defined and developed VERITAS Virtual User Models are available online in VERITAS repository [38].

#### 2.2.5. Evaluation Methodology of the Parameterized Cognitive Virtual User Model

Once the final Cognitive Virtual User Models were defined and developed, they were applied for accessibility assessment of a real application. A GUI-based Remote Health Monitoring application was used to that end [39]. The Health Remote Monitoring Application used for test the proposed models is a 2D tool that aims at empowering older people to personally handle their own health and wellbeing. This tool was selected to test the generated models and make the accessibility evaluation of developed 2D interfaces using the VerSim-GUI, because it fits the requirements of a Personal Health Care Application (e.g., health self-management, share information with caregivers/specialists, *etc.*) which is the center application of the models and tools presented on this study evaluation. Virtual user models’ simulations were focused mainly on older people (over 55), who were classified into three main categories:

- People who are healthy and, in most cases, can still lead busy and active lives, but who have just started to experience slight deteriorations in their quality of life due to ageing.
- People who are healthy, but are more likely to experience mild cognitive and physical problems due to ageing.
- People who are very likely to experience cognitive and physical deteriorations due to ageing.

Several simulations were done using various user models and usage cases, with the purpose of assessing the accessibility of the Remote Health Monitoring solution using the final designed and parameterized Cognitive Virtual User Models. Three main groups of potential users were selected to cover all the possible affected disabilities. These groups were: (1) older users; (2) users with low vision; and (3) users with motor problems (e.g., disabilities in: speed, dexterity, fatigue resistance, gait, fine motor skills muscle strength, *etc.*). Additionally, three main user models and several variations thereof were created using the VERITAS User Model Generator (VerGen) tool [40,41]. Table 1 shows the definition of the three main user models utilized for simulations.

The main tasks of the Health Remote Monitoring application were simulated for each of the virtual user models (*i.e.*, Edit profile, Take a measurement and Check medication calendar) using the VerSim-GUI (VERITAS GUI Simulation Viewer) tool [40,41]. Simulations were performed for each virtual user model created. Additionally, user cases to be tested were defined for each main task of the application, such as:

- Edit health profile: the user goes into “My ID card Menu”, selects “modify profile”, changes the “gender” (from male to female or vice versa), and “saves” the modification.
- Take measurement (blood pressure): the user goes to “My Health Status Menu”, selects “measure Blood pressure”, read the instructions to use the blood pressure sensor, “starts” the measurement and waits for the results.
- Check Medication Calendar: the user selects the option “Medication Calendar” from the main screen, reads the information about the schedules and dosage of each medication, and presses “ok”.

## 3. Framework Results

The proposed framework consists of three principal elements that together comprise the structure of final models. First, the Abstract User Model is constructed by the integration of existing models, medical studies, guidelines, methodologies, real user measurements in real conditions, and existing practices, user needs, as well as known accessibility guidelines and standards. The second element is the ACT-R framework, which links up the parameter values with each ACT-R module and with the different cognitive processes. Finally, the third element is task model implementation, represented by users’ performance of specific tasks and interactions.

### 3.1. Cognitive Abstract User Modeling Representation

Most relevant cognitive functions were investigated and described by dividing them in two major groups: basic and higher-level functions. Table 2 shows the specific cognitive functions of each group and their short descriptions. All of these cognitive functions affect elderly people, Parkinsonian and Alzheimer’s patients in different ways. For instance, reaction time slowing, deficits in different memory types, orientation, speech and language deterioration, difficulty in suppressing a prepotent response (Parkinson’s), *etc.*

As described previously, a table structure that relates the cognitive disabilities with the affected task models was also defined. The main information included in the cognitive AUM description table is: ICD classification, short pathology description, and parameters based in literature survey for every disability. The disability categories studied were: cognitive ageing, Alzheimer’s disease, Parkinson’s disease, hearing impairment, blind and low vision impairment, and speech impairment.

**Figure 3:**
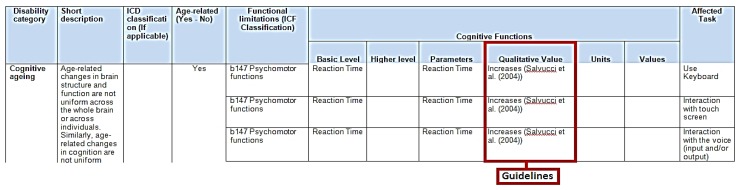
Enriched Cognitive AUM. Note: Salvucci *et al.* [42].

Some of the identified cognitive parameters contained only qualitative values. In order to enrich the cognitive AUM and to provide additional information and guidelines to the developers, these qualitative values were added to the cognitive AUM definition. An example of the final and enriched cognitive AUM definition table is presented in Figure 3.

### 3.2. ACT-R Cognitive Parameters and Modules

Related primitive tasks and cognitive process involved were defined for each ACT-R module as follows:

- Vision Module: *primitive tasks*: look (eyes), see (eyes), and focus (eyes); *cognitive process*: visual attention.
- Audio Module: *primitive tasks*: listening (ears)/attention; cognitive process: aural attention.
- Motor Module: *primitive tasks*: grasp, touch, reach, pull upwards, position, pull, hit and push (hands); *cognitive process*: procedural.
- Speech Module: *primitive tasks*: speak (voice); *cognitive process*: speech.
- Declarative Module: *primitive tasks*: memory; *cognitive process*: explicit memory.
- Goal and Imaginal Modules: *primitive tasks*: those related to procedural memory; *cognitive process*: procedural memory.
- Device Module: *primary tasks*: those related to the use of the mouse; *cognitive process*: visual attention.
- Procedural, Utility and Production compilation Modules: *primitive tasks*: decision making, procedural memory and safety decision; *cognitive process*: procedural (implicit) memory.

An ACT-R (sgp) User Model table was defined including the following information:

- The ACT-R module involved.
- The ACT-R (sgp) parameters, description, default values, and specific values for each defined cognitive impairment (cognitive Ageing, Alzheimer’s disease, Parkinson’s disease, hearing impairment, blind and low vision impairment and speech impairment).
- The affected primitive tasks per each ACT-R (sgp) parameter.
- The primary cognitive process involved (e.g., visual attention, reaction time, *etc.*)

**Figure 4:**
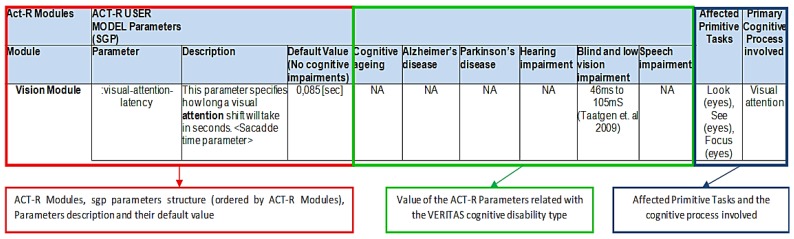
Structure of the ACT-R (sgp) User Model parameters. Note: Taatgen *et al.* [43].

Figure 4 shows an example with the structure of the ACT-R (sgp) user model parameters.

### 3.3. Task Model Implementation

Tasks within each disability, as related to the Personal Healthcare and Wellbeing sector, were collected and analyzed. A task was defined as a group of basic actions taken by a user, and it was subdivided into subtasks in order to define a closer link. A subtask was defined as a list of primary actions needed to perform a task. For example, the possible subtask for the task of configuring an application in a PC could be: selection of sound option menu, selection of vibration option menu, selection of language, *etc.*

A list of collected and analyzed affected tasks and subtasks, related to ICT solutions for the Personal Healthcare and Wellbeing sector, and for each cognitive function and disability category (Cognitive Ageing, Alzheimer’s disease, Parkinson’s disease, Hearing Impairment, Blind and Low Vision Impairment, and Speech Impairment) are presented in Table 3.

**Figure 5:**
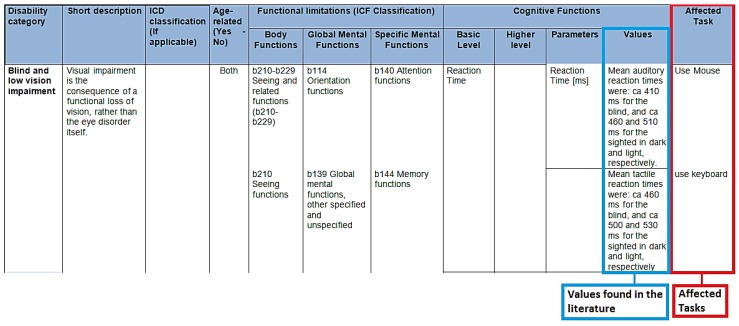
Cognitive AUM representation with affected tasks.

All collected information (task, subtasks, primitive tasks, cognitive state affecting primitive tasks, *etc.*) related to the Personal Healthcare and Wellbeing sector were organized into an affected tasks analysis table, which defines the link between application sectors, tasks, subtasks, and primitive tasks. This information was then merged in the cognitive AUM, in order to establish the link between the information from the affected tasks analysis and the information already contained in the cognitive AUM model (disabilities, metrics and parameters). An example of this association is the table presented in Figure 5. Empty fields in the table mean that quantitative values do not exist in the literature for the cognitive parameter directly related to the affected task.

### 3.4. Generic Cognitive Virtual User Model Representation

**Figure 6:**
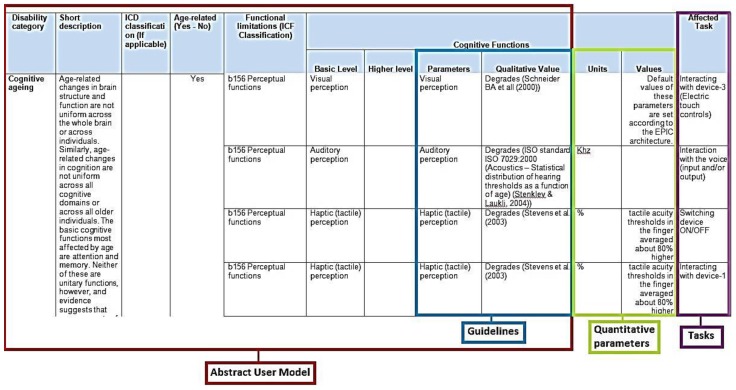
Structure of the Generic Cognitive Virtual User Model. Notes: Schneider *et al.* [44], Stevens *et al.* [45], Stenklev and Laukli [46].

**Figure 7:**
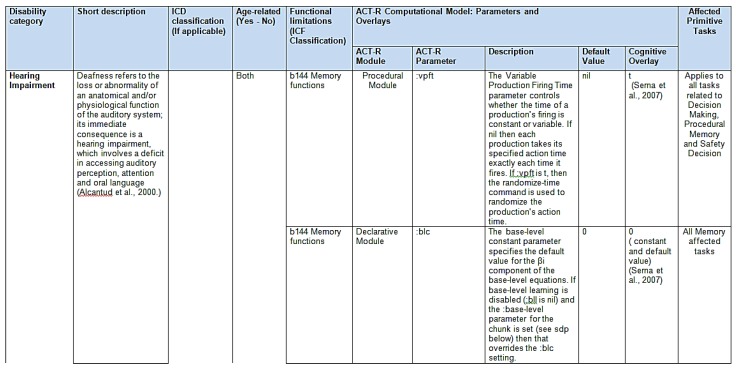
GCVUM with the ACT-R parameters. Notes: Alcantud *et al.* [47], Serna *et al.* [48].

As already hinted in Figure 2, the Generic Cognitive Virtual User Model merges together the information contained in the Cognitive AUM (quantitative and qualitative values), the ACT-R parameters values, the results of observation and measurements and the tasks derived from the task analysis tables. Figure 6 and Figure 7 present the final structure of the GCVUM and the GCVUM with the ACT-R parameters, respectively.

### 3.5. Representation of the GCVUM through Ontology

Ontologies were used to provide complete specification and representation of the final Cognitive Virtual User Model. The user models stored in the ontology includes: (1) the type of disability; (2) user capabilities; (3) user needs; (4) characteristics from cognitive user models; and (5) guidelines and standards.

**Figure 8:**
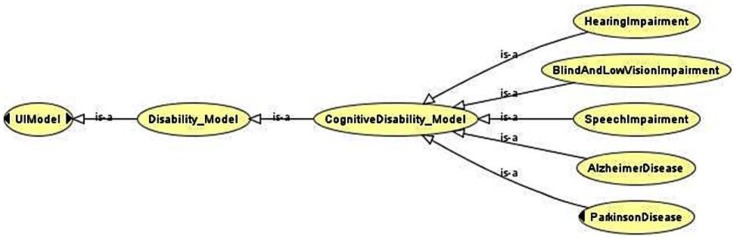
Ontology representation of the modeled cognitive disabilities.

Disabilities were designated as ontological classes. Each one of them involves and represents a cognitive disability inherited from the *CognitiveDisability_Model* class, as it is shown in Figure 8. The main objective of the superclass CognitiveDisability_Model is to define taxonomy as long as it does not define any property. The children classes define all the properties related to the particular disability.

**Figure 9:**
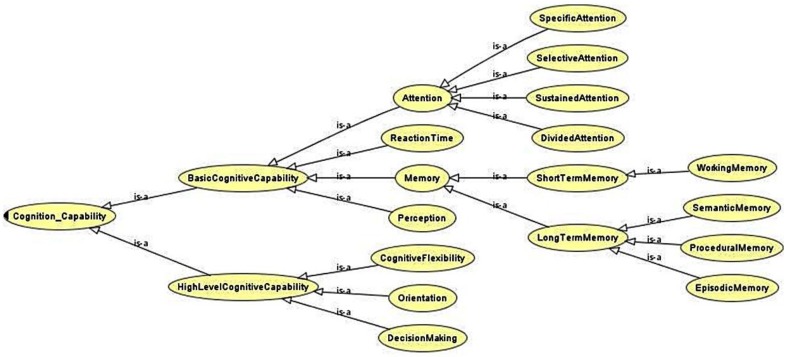
Cognitive capabilities affected by cognitive disabilities.

The affected cognitive capabilities are included and related to the cognitive disabilities. The superclass of this hierarchy is the class *Cognition_Capability*, represented in Figure 9. This class is again empty, and its only function is to define a new classification in the main ontology. The same pattern repeats for the rest of the classes within the hierarchy. Specifically, the leaf nodes have the property named *hasCognitiveMeasure*, which relates a cognition capability with its corresponding measure.

There are more than twenty modeled cognitive measures. Some of them are: working memory measure, orientation measure, episodic memory measure, haptic perception, missing information, *etc.* Each one is related to their corresponding cognition capabilities. The relationship is one to one in some cases and one to many in other cases. For example, for the class *Perception* the cognition capability class has three cognitive measures: auditory perception, haptic perception and visual perception.

The final representation of the GCVUM during the simulation was done using USer Interface eXtended Markup Language (UsiXML) [48], as the simulation platform uses a XML representation based on the USI-XML standard. This implies that the ontologies were transformed into UsiXML schemas in order to carry out the user model simulations. Due to the complexity and length of the ontology, it is not included in this document, but it is available at the VERITAS web site [30,49].

## 4. Evaluation through Simulation Results

### Evaluation of the Parameterized Cognitive Virtual User Model Results

User validation tests were carried out using three VERITAS tools; the Virtual User model generator (VerGen), the Virtual Editor VerSEd-GUI, and the simulation Editor (VerSim-GUI) [40,41]. Results of the accessibility assessment of the Remote Health Monitoring solution using the final designed and parameterized Cognitive Virtual User Models are described in this section. Different simulations were done using various user models and use cases, as already mentioned in the methodology section. Different test simulations were also performed for each virtual user model by loading the virtual user model, performing the tasks and using the application with the defined parameters.

Several simulations were done using the three main groups of potential users: (1) people with usual cognitive and physical deteriorations due to ageing; (2) people with visual impairments; and (3) people with motor impairment. The obtained results are presented in the following figures. In the figures the images on the left hand side provide the task durations (red bar) compared to the optimal user (green bar). Images on the right hand side show the graphical feedback on the interactions of the user with the UI, specifically the clicks inside or outside the predefined hot areas, and the itinerary of the mouse during the interactions. Mouse itinerary during the interactions was represented by a blue path for the optimal user and a green dotted path for the performance of tested user. Yellow dots are wrong clicks, red dots are actual clicks, and purple dots are optimal clicks. The analyses of these results for the three types of users are presented below.

#### User 1: Elderly

User 1, as stated before (Table 1), has the characteristics of a normal/standard old user: normal vision, normal hearing, normal cognitive status, and motor disabilities of an elderly person.

**Figure 10:**
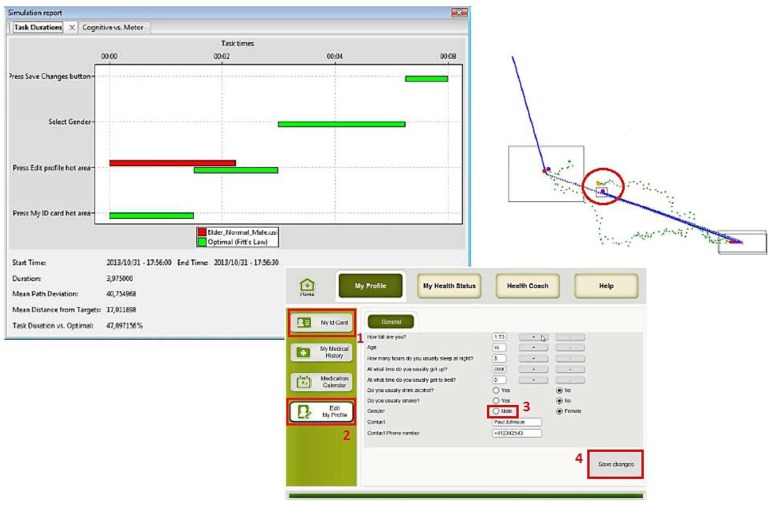
User 1 Cognitive VUM simulation: Edit health profile.

**Figure 11:**
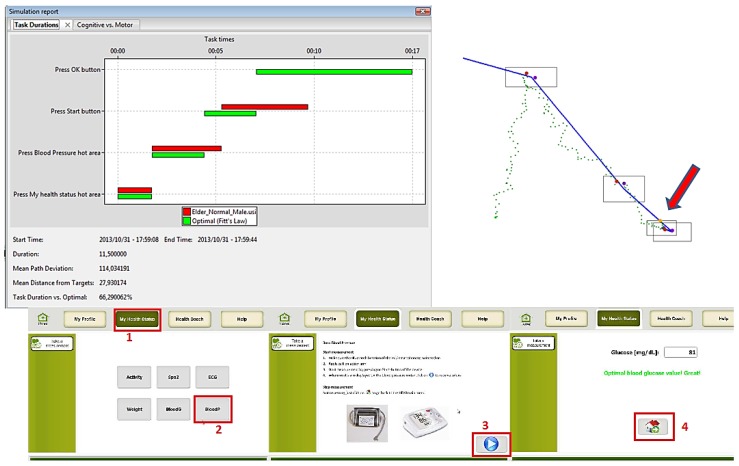
User 1 Cognitive VUM simulation: Take Measurements.

**Figure 12:**
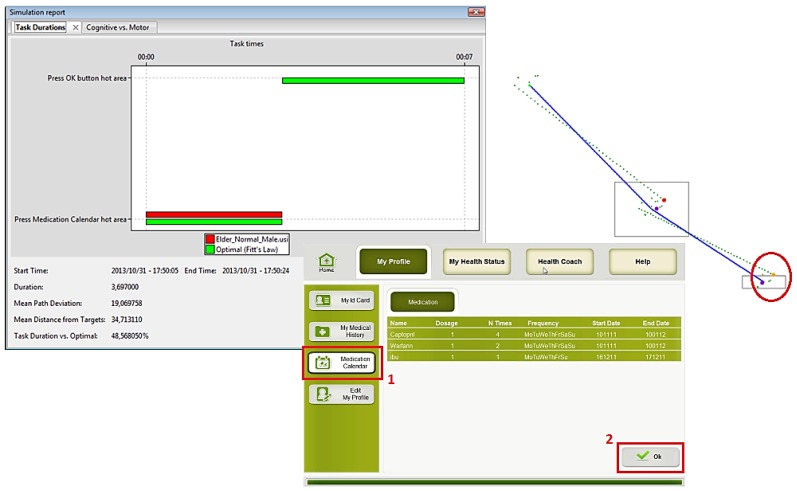
User 1 Cognitive VUM simulation: Check medication calendar.

Figure 10, Figure 11 and Figure 12 show the results of User 1 simulation. Results indicate that an older normal/standard user did not succeed in completing all requested tasks. The performance of the required tasks presented, in some cases, an execution delay or were not completed at all, as compared to the optimal user (green bars). For example, as Figure 10 shows, the user was not capable to press the “My ID card” hot area and neither selects the gender and save the changes (absence of the red bar). In the graphical simulation we can notice when the user failed to press the gender button (yellow dot, indicated with red circle), and also the irregular mouse trace that the user did to complete the task. In the same way Figure 11 and Figure 12 show some user errors during the simulation of “Take measurement” and “Check medication calendar” tasks. These results provide the designers the implicit recommendation of working on a better definition of hot areas, and being permissive to user errors or interaction duration.

Results confirm the lack of accuracy of elder VUMs in pressing and releasing the mouse button in the correct hot area. Table 4 shows these results.

#### User 2: Low Vision

**Figure 13:**
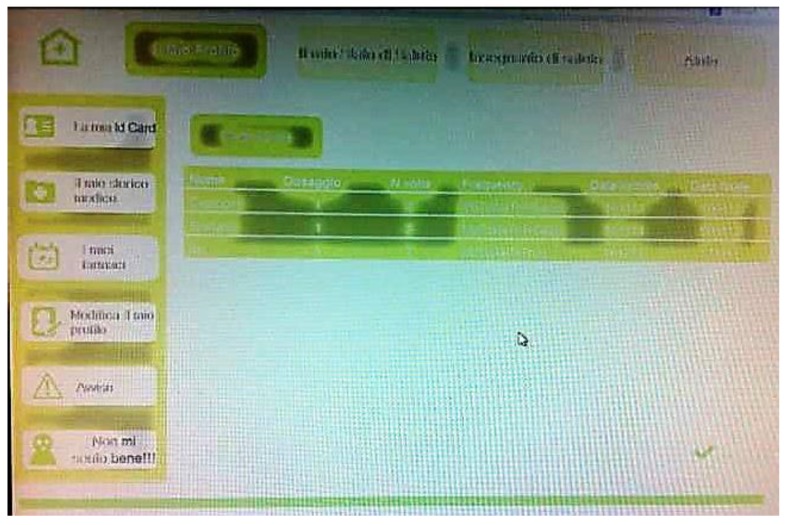
UI visual perception of User 2 using the application.

As shown in Table 1, User 2 has the characteristics of a person with a vision disability, specifically cataracts. In this case, simulation shows that visual perception of the user is obviously critically deteriorated. The UI appearance is clearly barely visible, as shown in Figure 13. The upper and side menus are not clear, texts are difficult to read, and buttons and icons are blurred. The content of the main (central) panel is also dispersed; buttons’ borders cannot be distinguished, and contrast is insufficient. With these results it is obvious that the design is not appropriate for a person with severe low vision problems. Even though some users (depending on the level of the visual impairment) might be able to complete some of the tasks, they would probably have a hard time understanding the provided content.

**Figure 14:**
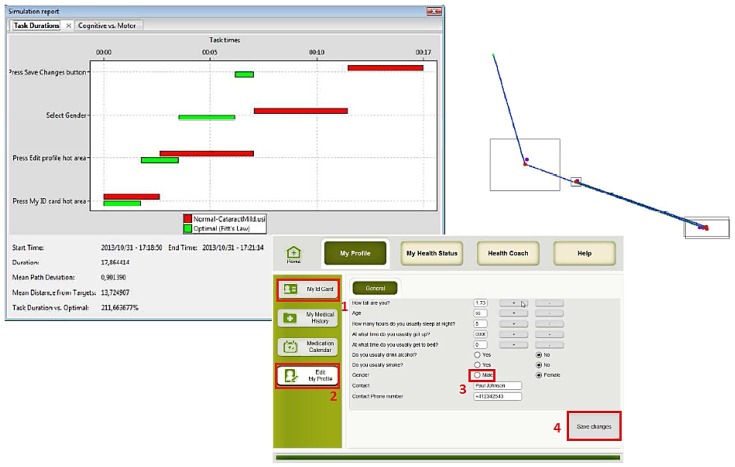
User 2 Cognitive VUM simulation: Edit health profile.

Results also show that, from the viewpoint of interaction, this user is capable of completing the required tasks. More precisely, simulations of the three use cases showed that the user was able to perform the tasks, but with a slight delay in all the cases. Figure 14 shows an example of this conclusion, specifically for the Edit Health Profile use case. The performance of all the required tasks presented an execution delay (red bars), as compared to the optimal user (green bars).

#### User 3: Motor Impairment

Finally, User 3 was created in order to get an insight into what interaction problems could be experienced by a user with motor problems. For this purpose, Parkinson disease was selected as the main feature. Several variations of this cognitive VUM were tested, basically changing the configuration of certain parameters.

**Figure 15:**
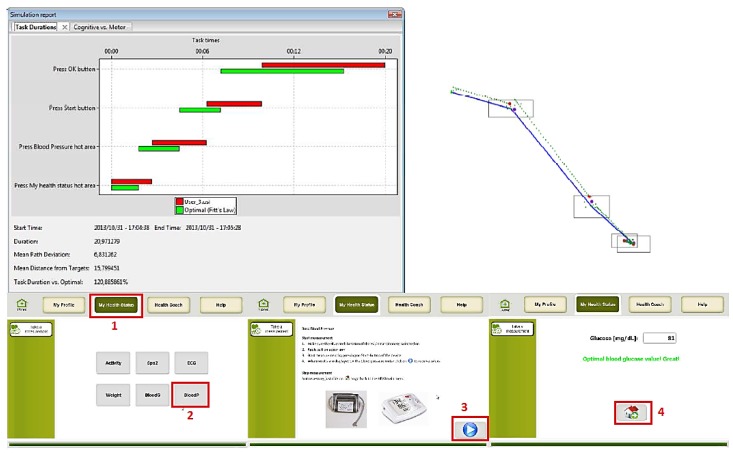
User 3 Cognitive VUM simulation: Take measurements (blood pressure).

First, a simulation with a “default” Parkinson user was done. Results show that even though the task duration is significantly increased, in comparison to the optimal user, User 3 completes the tasks successfully. However, from the graphical feedback on the interactions of the user with the UI, we can infer that some hot areas would need to be enlarged in order to assure successful interactions. An example of these results is presented in Figure 15, specifically for the Take Measurements use case.

**Figure 16:**
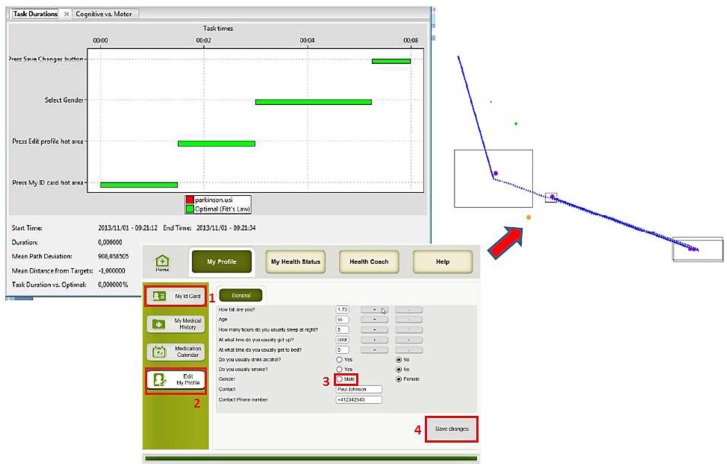
Modified User 3 Cognitive VUM simulation: Edit health profile.

At last, variations in the configuration parameters such as: wrist extension, fingers extension, *etc.*, were done. These variations provided significant differences in the results. For instance, Figure 16 shows a simulation where the user fails in performing the required task (Edit Health Profile). The tested user was no able to complete the task because clicks were performed out of the buttons’ predefined hot area (yellow dot). These results give the designers valuable information. For example, it is important to enlarge some hot areas by designing bigger buttons, increasing (clearly delimitating) the distance between hot areas, in order to avoid errors, and improve the interaction (e.g., when radio buttons are used, making the corresponding text clickable as well).

As a summary, Table 4 compiles the results of simulations. For each task, three parameters are shown: accuracy, number of events, and duration. All these parameters have been extracted from the simulation reports generated by VerSim GUI tool [41]. It is important to remark that this table compiles the results of the most restrictive simulations, where the error rate accepted is cero (hard fail in all events, including mouse presses and releases). This means that any attempt of mouse click that is not included in “optimal user’s” performance was considered as a “task failed” event.

Task fails in simulations do not imply that a real user would not be able to complete the task at all, but indicates that the user would probably have difficulties. With this feedback designers are able to improve and assess the level of accessibility of the application.

## 5. Discussion and Conclusions

Developers generally test their prototypes in terms of functionality, but without being able to systematically test them in terms of accessibility. As a result of the present study we conclude that, to overcome this undesirable situation, the design process of products and services for this market sector should aim to fulfill the following fundamental conditions: (1) Design the mainstream features for the widest possible range of capabilities; (2) Ensure that the service or application is adaptable to additional types of capabilities; and (3) Guarantee the connection with user interface devices.

The present approach represents a valuable tool for the development of accessible and usable ICT products and systems in general and specifically for health solutions. Designers and developers will be able to get the advantages that this tool provides to the design process, such as: (1) understanding user’s needs and characteristics through the user models; and (2) improving the application’s UI and the overall user experience, using the simulation results of user model interaction with their applications. As a result of the present study, along with the overall analysis done with other applications within the VERITAS project, we conclude that the parameterized virtual user models defined and tested managed to be a near real virtual representation of people affected by aging- and disability-originated functional impairments and cognitive states, as well as represent a useful tool to evaluate usability during the design, development and evaluation process of 2D application interfaces of ICT applications.

The modeling of virtual users with disabilities and different cognitive impairment states still remains a very challenging task, which has not been yet fully addressed. The parameterization of user cognitive models could help in achieving this goal, by allowing ICT researchers, designers, and developers to include and consider in their designs the cognitive status and perception processes of people with aging- and disability-derived functional impairments. Also the use of multisensorial techniques to train the virtual parameterized user models based on real user measurements in real conditions represents a valuable innovation in the design of more disability-friendly applications. This would help ease the road for providing efficient and accessible ICT solutions suitable for the particular medical conditions, needs, and capabilities of this important sector of the population based on real sensed measurements [15,27].

### Limitations and Future Work

The parameterization of the cognitive virtual user models has been accomplished in this work through the creation of a relationship between the ACT-R parameter values, the related primitive tasks and the cognitive processes involved. These cognitive attributes and parameters were selected through a comprehensive analysis of the standards, rules and guidelines found in the literature. Although good quality results have already been obtained on the basis of these data, it would be convenient to conduct wide-ranging research in collaboration with psychologists and users, in order to further improve the identification and definition of the cognitive values and their relation to different tasks. Additionally, parameter values could be extended to other cognition processes and primitive tasks in order to use this tool in the accessibility design and evaluation of 3D applications and systems.

Another limitation of the present cognitive virtual user models is the fact that they were designed only for the initial phases of Alzheimer’s and Parkinson’s diseases. This means that the values used to parameterize the user models are only related to the cognitive processes and characteristics of users in early stages of these specific diseases. For future assessments we propose to make an extension of this parameterized user models for more advanced stages of the diseases, adding the related cognitive and physical disabilities values for these stages.

The present study only aims to develop and evaluate the use of cognitive virtual user models during the design process of an ICT application and its accessibility evaluation. We have only centered our study in the evaluation of the usability or systematic use of an application interface. In order to have a better assessment of the virtual user models developed, the evaluation of cognitive virtual user models use by designers (e.g., application designers) during the design and developing process, and the testing of the designs with real disabled users is needed to make statistical comparisons between the results obtained from developed models and real users interactions with ICT applications.

## Figures and Tables

**Table 1:** Virtual user models created to test accessibility.

User Type	User Name	Motor Disability	Vision Disability	Hearing Disability	Cognitive Disability
User 1: Elderly	Elder motor	Elder	Normal	Normal	Normal
User 2: Low vision	Cataract 50	Normal	Cataract	Normal	Normal
	Glaucoma 50	Normal	Glaucoma	Normal	Normal
User 3: Motor impairment	Parkinson 50	Parkinson	Normal	Normal	Normal
	Parkinson 90				

**Table 2:** Basic and higher-level cognitive functions.

Basic Functions	Higher-Level Functions
**Reaction Time**	**Decision Making**
*Measure of the overall cognitive performance speed.*	*Selection of a belief or a course of action among several alternative possibilities.*
**Attention**	**Orientation**
*Involved in virtually all cognitive tasks. * *Subdivided in: selective, divided, and sustained.*	*Awareness of three dimensions: time, place and being.*
**Memory**	**Speech and Language**
*Ability to store, retain, and recall information. * *Subdivided in: semantic, episodic, procedural, and working.*	*The faculty or act of expressing thoughts, feelings, or perceptions by the articulation of words.*
**Perception**	**Cognitive flexibility**
*Recognition and interpretation of sensory stimuli. * *Subdivided in: visual, auditory, and haptic.*	*Ability to switch attention from one aspect of an object to another.*

**Table 3:** Affected tasks, subtasks and primitive tasks.

Task	Subtasks	Primitive Tasks
▪ Using PC ▪ Using mobile devices ▪ Interfacing with medical devices ▪ Using wrist worn device interface ▪ Contact emergency services ▪ Using sensors	▪ Switch on/off PC ▪ Switch on/off screen ▪ Handling mouse ▪ Use keyboard ▪ Switch on/off cellphone ▪ Handling device ▪ Interaction with touch screen ▪ Interaction with the voice (input and/or output) ▪ Interaction with the sound (input and/or output) ▪ Switching device on/off ▪ Interaction with device ▪ Switch service on ▪ Putting sensors on/off ▪ Interact/Handling with sensors	▪ See (eyes) ▪ Pointing (eyes, hands) ▪ Reach (hands) ▪ Push (hands) ▪ Pulling (hands) ▪ Move (hands) ▪ Touch (hands) ▪ Grasp (hands) ▪ Turn (hands) ▪ Click (finger) ▪ Press (finger and/or stylus pen) ▪ Talk (hands, voice) ▪ Speak (mouth) ▪ Hear (ears)

**Table 4:** Overall results of simulations with Virtual User Models (VUMs).

		Elder		Low Vision		Motor Impairment	
		Elder Motor 50	Elder Motor75	Cataract 50	Glaucoma 75	Parkinson 50	Parkinson 90
**Check Medication**	**Accuracy**	0,25	0	1	1	0	1
	**Duration (sec.)**	34	Failed	154	57	Failed	45
	**N° events**	404	Failed	295	295	Failed	365
**Take a measurement**	**Accuracy**	0	0	1	1	1	1
	**Duration (sec.)**	Failed	Failed	212	78	62	68
	**N° events**	Failed	Failed	412	412	527	546
**Edit profile**	**Accuracy**	0	0	1	1	0	0
	**Duration (sec.)**	Failed	Failed	231	68	Failed	Failed
	**N° events**	Failed	Failed	343	343	Failed	Failed

Note: *Accuracy*: indicates the success rate of each VUM: 0 = task not completed, 1 = task steps completed correctly; *Duration*: duration in seconds to perform and complete the task; *N° events*: total amount of mouse movements, presses and releases; only showed for users that completed the task successfully.
